# The effectiveness of different education methods conducted at different times for decreasing the time required for a parturient to position herself for epidural placement

**DOI:** 10.1186/s12884-022-04879-6

**Published:** 2022-07-11

**Authors:** Chen Yang, Yan Cheng, Jianying Hu, Yaojun Lu, Xinhua Yu, Shaoqiang Huang

**Affiliations:** 1 Department of Anesthesiology, Obstetrics & Gynecology Hospital, Fudan University, 128# Shenyang Road, 200090 Shanghai, China; 2 Division of Epidemiology, School of Public Health, Biostatistics and Environmental Health, University of Memphis, TN Memphis, USA

**Keywords:** Labour analgesia, Photo education, Video education, Body position, Advance education

## Abstract

**Background:**

Decreasing the anaesthesia preparation time for primiparas experiencing painful uterine contractions is clinically relevant. This prospective intervention study investigated the effect of various educational methods conducted at different times on body positioning for primiparas undergoing labour analgesia.

**Methods:**

Ninety primiparas who were about to receive labour analgesia were randomly divided into a verbal instruction group, a photo instructions group, and an educational video group for immediate education, and 60 primiparas who were willing to receive labour analgesia but were not in labour were randomly divided into a photo instruction group and an educational video group for advance education. The times required for body positioning were compared.

**Results:**

In the immediate education cohort, the body positioning time in the verbal group (50.48 ± 28.97 s) was significantly longer than those in the photo group (30.47 ± 6.94 s) and the video group (23.14 ± 9.74 s) (*P* = 0.00). In the advance education cohort, the time in the photo group (17.47 ± 6.48 s) was longer than that in the video group (13.71 ± 7.01 s) (*P* = 0.042). Whether photos or videos are used, advance education can significantly decrease body positioning time.

**Conclusions:**

Video or photo education for primiparas who are about to receive labour analgesia can decrease the body positioning time and is more effective when provided in advance.

**Supplementary Information:**

The online version contains supplementary material available at 10.1186/s12884-022-04879-6.

## Background

Neuraxial block is currently recognized as the most effective form of labour analgesia with minimal effects on the mother and infant, and patient positioning is an important aspect of neuraxial block preparation [1]. Most women undergo an epidural puncture in the lateral decubitus position, with the exception of those with morbid obesity, who may choose to undergo epidural puncture in the sitting position. As primiparas suffer from painful uterine contractions, communicating effectively with medical staff is often difficult for them during the administration of labour analgesia [2]. Increased abdominal girth, bulky body, etc. complicate primiparas’ ability to position themselves, and anaesthesia preparation often takes longer [3]. In contrast to patients undergoing elective caesarean section, decreasing the anaesthesia preparation time is more meaningful for primiparas experiencing labour pain.

The most effective strategy to decrease the anaesthesia preparation time is primiparous education. Currently, the most commonly used strategy in clinical practice is verbal education immediately before labour analgesia, the effectiveness of which varies markedly from individual to individual. Furthermore, studies have shown that people generally remember 10% of what they read, 20% of what they hear, 30% of what they see, and 70% of what they hear and see. Thus, information received through photos and videos is more easily remembered [4]. We speculate that the use of photos or videos to educate primiparas may decrease the epidural anaesthesia preparation time. However, the magnitude of clinical significance is unknown. Additionally, since primiparas who received education are a special group experiencing labour pains, whether education in the early labour process without obvious contractions is better is uncertain. Therefore, we designed this prospective intervention study to investigate the influences of different means and timing of education on the positioning of primiparas undergoing labour analgesia administration.

## Methods

This study was approved by the China Ethics Committee of Registering Clinical Trials (ChiECRCT20200030) and conducted at the Obstetrics and Gynecology Hospital after signed consent forms were obtained from participating parturients. The trial was registered prior to patient enrolment at the Chinese Clinical Trial Registry (ChiCTR1900025231). This manuscript adheres to the applicable CONSORT guidelines.

The inclusion criteria were primiparas with an American Society of Anesthesiology (ASA) score of II and a singleton and term pregnancy who were willing to undergo labour analgesia in the labour room. The exclusion criteria were contraindications to neuraxial anaesthesia; communication barriers precluding cooperation with the study protocol; difficulty moving and requiring positioning assistance, such as for separation of the pubic symphysis; and previous experience with neuraxial anaesthesia or working as medical staff. Primiparas who had been included in the study were excluded from the analysis in any of the following cases: primiparas who did not undergo labour analgesia due to reasons such as emergency delivery or transfer to an emergency caesarean section and primiparas who had difficulties with positioning themselves and required assistance from others.

The clinical trial consisted of two strata for the timing of education (Fig. 2). The first stratum included ninety primiparas who had been in labour and were about to undergo labour analgesia. One envelope was randomly selected from three envelopes containing a note with verbal, photo, or video written on it, and the primiparas were randomly divided into a verbal instruction group, a photo instruction group and an educational video group according to the contents of the selected envelope. Education on anaesthesia positions was carried out between intermittent painful uterine contractions, and anaesthesia position preparation was started immediately after the education session. These groups of primiparas were the immediate education cohort.

The second stratum included sixty primiparas who were willing to receive labour analgesia but had not yet gone into labour. One envelope was randomly selected from two envelopes containing notes with either photo or video written on it. The primiparas were randomly divided into a photo instruction group and a video instruction group according to the contents of the selected envelopes, and then education on anaesthesia position was carried out. The neuraxial block was not performed until the labour analgesia condition was met. These groups of primiparas were the advance education cohort.

Video instruction group: A customized epidural anaesthesia education video (10 s) described the process of body positioning, and subtitles emphasized the key points (Video 1).

**Fig. 1:**
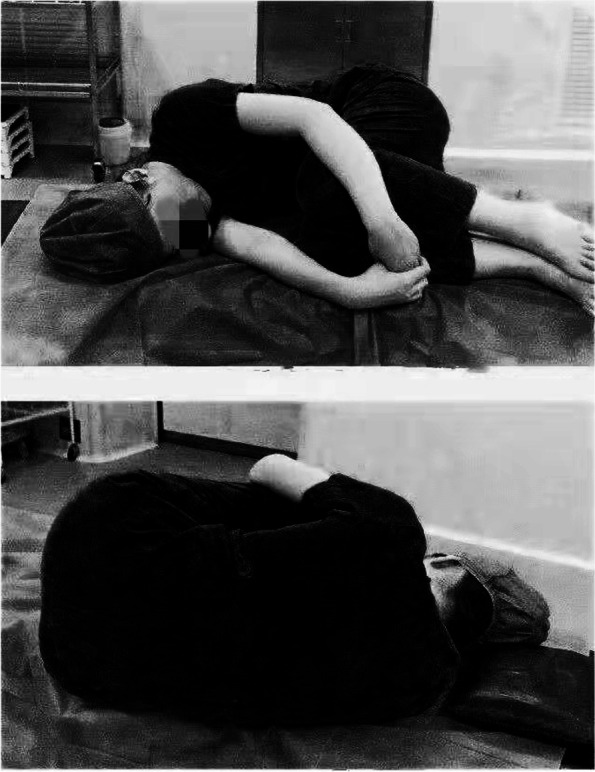
Educational pictures. The words in the picture: turn your back to the doctor, and move to the bedside at the same time, your back is perpendicular to the bed; bend your knees with your thighs close to your abdomen; and bend your head to your chest as far as possible

Photo instruction group: Preparation of epidural anaesthesia body position photos: white cardboard (20 cm × 30 cm) was used, which had a photo of the correct anteroposterior position for neuraxial anaesthesia and a photo of the correct lateral position for neuraxial anaesthesia, both of which were laminated (Fig. 1).

Verbal instruction group: Generalized and standardized narrative language was used: turn your back to the doctor and move to the bedside; bend your knees with your thighs close to your abdomen; and bend your head to your chest as far as possible so that your back and lower back bend into an arc shape and, at the same time, your back is perpendicular to the bed.

After the primiparas entered the labour room, they were assessed and grouped by the anaesthesia nurse in charge of the study. The general condition and pain score assessed by a visual analogue scale (VAS: 0 cm, no pain; ~ 10 cm, painful) of each primipara were recorded, and the primiparas were educated according to the assigned group. The anaesthesiologist in charge of recording the time stood at the bedside near the head of the primipara when anaesthesia preparation began to record the body positioning time while ensuring the safety of the primipara. Then, a senior anaesthesiologist in charge of the anaesthesia operation evaluated the position. If the position was not satisfactory, the primipara was readjusted, but the readjustment time was not counted as a part of the body positioning time. Subsequently, routine analgesic procedures were performed, and the study was concluded.

The primary observation outcome was body positioning time, i.e., the time from turning the body to completion of all movements as perceived by the primipara. All body positioning started at the end of a uterine contraction, and the primipara was told that if a contraction occurred during postural movement, the primipara should immediately signal or inform the anaesthesiologist who was recording the time; postural movement was suspended, and time recording was paused. After the uterine contraction ended, the primipara was signalled to continue to move, and the time recording was continued; the number of pauses and the number of cases were recorded. The secondary observation outcome was satisfaction of the anaesthesiologist with the body position; the evaluation included four points, i.e., holding the knees with both hands such that the thighs are close to the abdomen, bending the head to the chest as far as possible, bending the back and lower back into an arc shape, and making the back perpendicular to the bed. If all our points were satisfied and no further adjustment was needed, the position was defined as satisfactory; if 1 point was not satisfactory and local readjustment was required, the position was defined as fair; if 2 points or more were not satisfactory and major adjustment was required, the position was defined as unsatisfactory. In addition, patient satisfaction with the postoperative analgesia was evaluated as 1 (very dissatisfied), 2 (dissatisfied), 3 (neither dissatisfied nor satisfied), 4 (satisfied), or 5 (very satisfied) when they left the labour room.

### Statistical analysis

Because no relevant data regarding positioning time are available from previous studies for reference, we used a preliminary test to determine the sample size. In the preliminary test, for 10 primiparas given labour analgesia who received immediate education via verbal instructions, 10 primiparas who received immediate education via photo instructions, and 10 primiparas who received advance education through photo instructions, the measured positioning times were 53.73 ± 36.28 s, 30.4 ± 16.95 s, and 17.35 ± 6.51 s, respectively. Using two-tailed tests (α = 0.05 and β = 0.1), a comparison of education timing was conducted on advance education vs immediate education via photo instruction, and a minimum of 23 women were calculated to be required in each group. Then, using two-tailed tests (α = 0.05 and β = 0.1), to compare educational means within the immediate education groups with verbal or photo instructions, only 15 women were needed for each comparison considering the possibility of 20% dropout. Therefore, we aimed to recruit 30 women in each group (five groups in total), resulting in a total of 150 women.

Statistical analysis of the data was performed using SPSS 11.5 software (SPSS Inc., Chicago, IL, USA). Measurement data are expressed as the mean ± standard deviation. Count data were analysed using the chi-square test, and rank data were analysed using the Wilcoxon rank sum test. Differences between groups were analysed using variance analysis, and if a difference between groups was statistically significant, the least significant difference (LSD) test was used to perform pairwise comparisons between groups.

Given the nested structure of trial design, we first presented results for each education timing arm, compared education methods within each education timing arm and across education timing arms for each educational methods, and then employed a general linear mixed model to examine the effect of education methods by the body mass index (BMI) and age of the primiparas in both arms, with adjustment for the fact that education methods are nested within education time. The correlation between the education of primiparas and the body positioning time was analysed using Pearson’s correlation analysis. *P* < 0.05 was considered statistically significant.

## Results

**Fig. 2:**
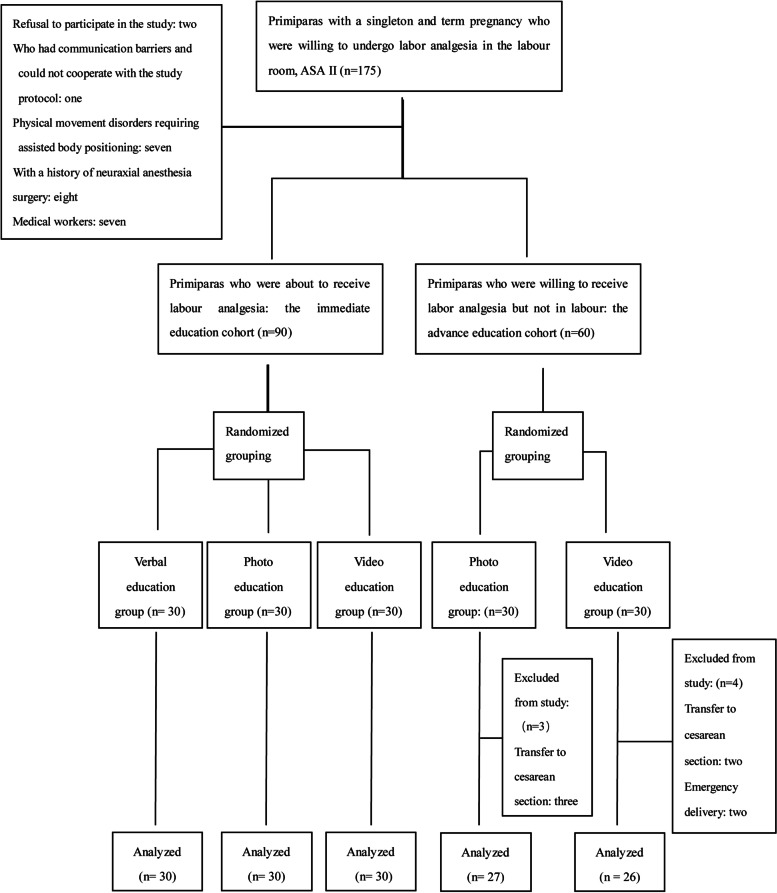
Consort flow chart of recruitment

One hundred seventy-five primiparas were initially recruited in the study, and 25 were excluded based on the exclusion criteria. Eventually, 150 primiparas were included. Of those included, 90 primiparas were about to receive labour analgesia, and 60 primiparas were not yet in labour. After each cohort was randomly grouped, all 90 primiparas in the immediate education cohort completed labour analgesia and were included in the final analysis, while 7 primiparas in the advance education cohort did not undergo labour analgesia; therefore, 53 women in the advance education cohort were included in the final analysis (Fig. 2).

The general conditions of the primiparas in each group are shown in Table 1; no significant differences were found between the groups (*P* > 0.05).

**Table 1:** Comparison of the general conditions of primiparas in each group

	Immediate education			Advance education	
	Verbal *n* = 30	Photo *n* = 30	Video *n* = 30	Photo *n* = 27	Video *n* = 26
Height (cm)	162.9 (4.4)	162.8 (3.6)	163.6 (6.3)	163.7 (3.8)	163.1 (5.6)
Weight (kg)	70.1 (16.7)	70.7 (7.2)	73.3 (10.2)	74.1 (13.8)	69.0 (11.6)
BMI(kg/m^2^)	26.3(6.1)	26.8(3.3)	27.4(3.6)	27.6(4.7)	26.0(3.7)
Age (yr)	28.8 (3.4)	30.7 (3.2)	30.0 (3.5)	29.2 (3.2)	30.0 (2.9)
Gestational age (weeks)	39.3 (1.1)	39.4 (1.2)	39.4 (1.7)	39.6 (1.2)	39.5 (1.2)
Cervical dilatation (cm)	2.1 (0.3)	2.4 (1.1)	2.4 (0.8)	0.5 (0.8)	0.4 (0.50)
Educational background (High school and below/the University/Postgraduate)	4/25/1	3/25/2	3/26/1	2/23/2	3/19/4

Values are mean (SD)

In the immediate education cohort, the body positioning time was significantly shorter in the photo group (30.47 ± 6.94 s) than in the verbal group (50.48 ± 28.97 s) (*P* = 0.00) and shorter in the video group (23.14 ± 9.74 s) (*P* = 0.00); the body positioning time was not significantly different between the photo group and the video group. In the advance education cohort, the body positioning time was longer in the photo group (17.47 ± 6.48 s) than in the video group (13.71 ± 7.01 s) (*P* = 0.042). In the comparison between the two cohorts, the body positioning time for the photo group in the immediate education cohort was longer than that for the photo group in the advance education cohort (*P* = 0.003); similarly, the time for the video group in the immediate education cohort was longer than that for the video group in the advance education cohort (*P* = 0.005). Body positioning by three primiparas in the verbal group and one primipara in the photo group in the immediate education cohort was interrupted due to painful uterine contractions, while body positioning of the primiparas in the other groups was not interrupted. The number of pauses for all primiparas interrupted during body positioning was one. The comparison of VAS scores at the time of education revealed that the VAS scores for the primiparas in the photo group, 0 (0, 2), and the video group, 0 (0, 2), at the time of advance education were much lower than those for the primiparas in the photo group, 7 (6, 8), and video group, 8 (6, 9), at the time of immediate education (*P* = 0.00) (Table 2).

**Table 2:** Comparison of VAS scores at the time of education and at the time of body positioning of primiparas in each group

	Immediate education						Advance education				
	Verbal *n* = 30	Photo *n* = 30	Video *n* = 30	P1	P2	P3	Photo *n* = 27	Video *n* = 26	P4	P5	P6
Body positioning time (s)	50.48(28.97)	30.47(6.94)	23.14(9.74)	0	0	0.11	17.47(6.48)	13.71(7.01)	0.042	0.003	0.005
Pause in body positioning (case)	3	1	0	0.31	0.18	0.33	0	0	1	0.32	1
VAS scores	8(7, 10)	7(6, 8)	8(6, 9)	0.17	0.23	0.18	0(0, 2)	0(0, 2)	0.23	0	0

Values are mean(SD)or median (IQR[range])

Immediate education

P1: comparison between the verbal group and the photo group

P2: comparison between the verbal group and the video group

P3: comparison between the photo group and the video group

Advance education

P4: comparison between the photo group and the video group

P5: comparison between the photo group of the immediate education cohort and the photo group of the advance education cohort

P6: Comparison between the video group of the immediate education cohort and the video group of the advance education cohort

The results of the general linear model regarding the differences in body positioning between different groups showed significant differences in body positioning times between groups (*P* = 0.00) after controlling for the educational background, BMI, and age of primiparas. The relationship between BMI and the body positioning time of primiparas was statistically significant (*P* = 0.042) (Table 3).

**Table 3:** General linear model of the differences in body positioning times among different groups

	**Sum III squared**	**df**	**Average squared**	**F**	**P**
**Intercept**	50.255	1	50.255	0.135	0.714
	38,121.801	102.635	371.430		
**Group**	23,389.956	4	5847.489	15.945	< 0.001
	37,038.748	101	366.720		
**Educational background**	1354.946	2	677.473	1.847	0.163
	37,038.748	101	366.720		
**BMI**	1563.323	1	1563.323	4.263	0.042
	37,038.748	101	366.720		
**Age**	151.880	1	151.880	0.414	0.521
	37,038.748	101	366.720		

Pearson’s correlation analysis was performed for the body positioning time and educational background of the primiparas in various groups, but no significant correlation was found (*r*^2^ < 0.3).

In the comparison of the degree of satisfaction of anaesthesiologists with the body position of various groups, the degree of satisfaction of anaesthesiologists with the video group in the immediate education cohort was higher than that with the photo group in the immediate education cohort (z^2^ = -2.58, *P* = 0.005); additionally, the degree of satisfaction of anaesthesiologists with the video group in the advance education cohort was higher than that with the photo group in the advance education cohort (z^2^ = -2.00, *P* = 0.04). In the comparison between cohorts, the degree of satisfaction of anaesthesiologists with the video group in the advance education cohort was higher than that with the video group in the immediate education cohort (z^2^ = -2.64, *P* = 0.01), and the degree of satisfaction of anaesthesiologists with the photo group in the advance education cohort was higher than that with the photo group in the immediate education cohort (z^2^ = -2.23, *P* = 0.008) (Table 4).

**Table 4:** Comparison of the degree of satisfaction of anesthesiologists with body positioning in each group

satisfaction of the anesthesiologist	Immediate education			Advance education	
	Verbal *n* = 30	Photo *n* = 30	Video *n* = 30	Photo *n* = 27	Video *n* = 26
ksatisfactory	16 (53.5%)	18 (60%)	25 (83.3%)	18 (66.7%)	24 (92.3%)
fair	9 (30%)	10 (33.3%)	5 (16.7%)	9 (33.3%)	2 (7.7%)
unsatisfactory	5 (16.7%)	2 (6.7%)	0 (0)	0 (0)	0 (0)

Values are number(proportion)

## Discussion

Our study found the advance education using video or photo instructions before labour significantly decreased the body positioning time of primiparas. In the immediate education cohort, video and photo instructions also decreased the body positioning time of the primiparas compared a verbal instruction. Furthermore, the degree of satisfaction of anaesthesiologists with positioning was highest in the video group in the advance education cohort and lowest in the verbal group in the immediate education cohort.

Among information obtained by humans, 83% is derived from vision, 11% from hearing, 3.5% from smell, 1.5% from touch, and 1% from taste. In addition, people can generally remember 10% of what they read, 20% of what they hear, 30% of what they see, and 70% of what they hear and see [4]. Many studies have applied photos and videos for preoperative care and patient education, resulting in higher patient satisfaction and lower preoperative anxiety [5, 6]; furthermore, the prognosis of patients is better when photos and videos are used in education [7]. The application of video education in preoperative visits for anaesthesia not only improves patient satisfaction and reduces preoperative anxiety [8] but also significantly decreases the visit duration [9]; patients are better able to choose an anaesthesia method [10], and anaesthesiologists are also more satisfied with this method [11]. Thus far, no studies have explored the effect of video education regarding body positioning during anaesthesia, and only one study on providing video education regarding anaesthesia to primiparas undergoing elective caesarean section compared only preoperative anxiety and postoperative satisfaction among primiparas [12]; therefore, our findings fill a gap in this field.

Most primiparas who undergo epidural anaesthesia for labour analgesia experience painful uterine contractions, mental stress, and loss of physical strength for a period during immediate education. Even if photos and educational videos are more acceptable, many primiparas are still unable to concentrate or cannot apply the instructions [3, 12]. The data regarding paused body positioning also revealed that when body positioning took longer, the probability of encountering another contraction was higher. In this study, when body movement was paused, time recording was also paused. If this period of time was included, the body positioning time for the verbal education group would have been even longer, and the difference between the groups would have been even greater. In view of the particularity of primiparas undergoing labour analgesia, we studied education timing. We conducted advance education for primiparas who had not entered labour, and at that time, the VAS scores for the primiparas were much lower than those for the immediate education cohort, and the primiparas were almost not affected by uterine contractions. This study found that advance education, regardless of the format, significantly decreased the anaesthesia preparation time the primiparas and that the body positioning time of the primiparas in the video education group in the advance education cohort decreased to 13.71 ± 7.01 s.

Previous studies have found a correlation between patient educational background and the degree of understanding after education [13, 14]. In this study, no correlation was found between the educational background of the primiparas and the body positioning time for anaesthesia preparation in all groups. One reason for this result may be because the tension and anxiety resulting from the painful uterine contractions negated the gap between the learning ability of primiparas with different educational backgrounds. Photos and videos are easier to understand. In addition, the educational background of primiparas in our study were generally high; few primiparas had an education background of high school and below.

This study has limitations. First, no verbal group was included in the advance education cohort. For the primiparas who were not in labour, compliance with verbal education was very poor; therefore, this study group was not included. However, the results from the immediate education cohort confirmed that the body positioning time for the verbal instruction group was much longer than those for the video group and the photo group. Second, advance education was conducted only for the primiparas who entered the labour room and qualified for the study, but the time between education and the start of anaesthesia administration was not recorded and studied. If maternal education is advanced to the last antenatal examination, whether the long time will lead to forgetting and the impact on the efficiency of education need to be further studied. Third, to ensure the objectivity of the body positioning time, the medical staff did not provide any assistance when each primipara positioned her body. However, due to humanitarian reasons, for those primiparas who encountered difficulty when positioning their body by themselves, the medical staff assisted in positioning, and these primiparas were excluded from the analysis. In fact, all the included primiparas completed body positioning independently. Fourth, the findings obtained in this study were only for primiparas receiving labour analgesia in the lateral recumbent position, and further studies are needed to confirm whether the findings are applicable to primiparas having labour analgesia in the sitting position.

## Conclusion

In conclusion, advance video education for primiparas who are not in labour and are willing to receive labour analgesia can significantly decrease the body positioning time and improve anaesthesiologist satisfaction regarding body position; for primiparas who are about to receive labour analgesia, video or photo education can also be preferentially used to decrease the body positioning time and improve anaesthesiologists’ satisfaction regarding body position.

## Supplementary Information

**Additional file 1.**

## Data Availability

All authors had full access to the data and materials. The datasets used and/or analysed during the current study available from the corresponding author on reasonable request.
